# *Hermetia illucens* Protein Conjugated with Glucose via Maillard Reaction: Antioxidant and Techno-Functional Properties

**DOI:** 10.1155/2021/5572554

**Published:** 2021-04-24

**Authors:** Vusi Vincent Mshayisa, Jessy Van Wyk

**Affiliations:** Department of Food Science and Technology, Cape Peninsula University of Technology, Bellville 7535, South Africa

## Abstract

The food industry is considering novel sources of proteins with enhanced functionalities to meet the increasing demand and population growth. Edible insect proteins have emerged as an alternative that is environmentally friendly and economically viable and thus could make a significant contribution to global food security. This study was aimed to establish the effect of conjugation via the Maillard reaction on the antioxidant and techno-functional properties of black soldier fly larvae protein concentrate. Reaction mixtures containing black soldier fly larvae protein concentrate and glucose (2 : 1 weight ratio) were wet-heated at 50, 70, and 90°C for 2, 4, 6, 8, and 10 h, respectively, with an initial pH of 9. The results showed that the browning indices of the black soldier fly larvae-glucose (BSFL-Glu) model system increased with an increase in reaction time and temperature, with conjugates formed at 90°C exhibiting the highest browning intensity at 420 nm. At 50°C, the DPPH-RS of the conjugates ranged from 15.47 to 32.37%. The ABTS^+^ radical scavenging activity of BSFL-Glu conjugates produced at 90°C exhibited significantly (*p* < 0.05) higher scavenging activity as a function of reaction time. The foaming capacity of BSFL-Glu conjugates produced at 70°C showed a significant increase (*p* < 0.05) as a function of reaction time. Principal component analysis was applied to browning and antioxidant indices. Component 1 of the score plot accounted for 89%, while component 2 accounted for 8% of the observed variability and allowed discrimination/differentiation of the samples based on the heating temperature. These findings provide a practical means to improve the functionality of novel edible insect proteins for food application.

## 1. Introduction

The predicted population increase to 9 billion people by the year 2050 presents a considerable threat to global food security. This is exacerbated by climate change, energy crises, and the consumption and demand patterns for good quality protein for human consumption [1, 2]. Edible insects have been proposed as an alternative food protein source that can address the current and future nutritional, health, economic, and food security concerns [3–5]. With the growing interest in edible insect protein consumption, industrial farming of species such as mealworm larvae (*Tenebrio molitor*) and black soldier fly larvae (*Hermetia illucens*), which are high in protein content, has been expanding due to investment in research and development, leading to a stable supply, consistent quality, cost-effectiveness, and hygienic production. Proteins extracted from edible insects and used in food applications as functional ingredients (in powder form or paste) may have greater success in terms of acceptance for human consumption since the willingness to consume whole insects is low [6, 7].

Apart from the nutritional value, food proteins provide unique techno-functional properties which affect their behaviour in food systems during preparation, processing, storage, and consumption and contribute to the quality and sensory attributes of food. To further promote the application of edible insects proteins in food applications, physical [8], chemical [9], and enzymatic [10] methods have been explored to enhance their techno-functional properties. Due to the potential health hazards brought in by chemical modifications, most of these techniques cannot be applied in food processing. Furthermore, it is difficult to widely apply physical modification as it involves mechanical forces such as high pressure or shear [11].

In the last decade, the Maillard reaction—a non-enzymatic chemical reaction between a carbonyl compound (usually a reducing sugar) and protein amino groups—has been reported to remarkably improve the techno-functional properties of proteins and even endows them with novel functionality. The Maillard reaction (MR) leads to the formation of protein-sugar conjugates with the enhanced emulsifying ability [12], solubility, antibacterial, antioxidant properties [13, 14], and even alleviates the allergenicity of proteins [15–17]. As reported by Medrano et al. [18], glycation of *β*-lactoglobulin with glucose exhibited improved foaming stability, suggesting increased adsorption to the air/water interface. Conjugates with superior emulsion properties were also reported by Diftis and Kiosseoglou [19].

There is now comprehensive scientific evidence showing that protein techno-functionality can be substantially improved without the use of any chemical reagents by covalent coupling with saccharides via the MR, thereby making this technique safe. [20–23]. The rate and degree of the MR are influenced by various factors such as temperature, time, relative humidity (RH), pH, and the molar ratio of the reactants, hence the nature of the products produced and their functional properties [24]. Recently, various scientific publications have studied the effect of heating temperature on the functional properties of conjugates generated from fructose-lysine and ribose-lysine [25], coconut sap [26], bovine serum albumin-glucose [15], and whey protein isolate with xylooligosaccharide [13] model systems. Guérard and Sumaya-Martinez [27] reported that the antiradical scavenging effect was improved by 75% when casein peptone and cod viscera hydrolysate were heated in the presence of glucose. However, there is a paucity of information about the effect of conjugation on the antioxidant activity and techno-functional properties of edible insect proteins extracted from black soldier fly larvae (BSFL). Since the Maillard glycation between reducing sugars and amino acids/proteins is inevitable during thermal food processing and manufacturing, it is imperative to understand its effect on novel proteins extracted from edible insects.

Therefore, this study was aimed to investigate the effect of conjugation time and temperature on the antioxidant activity and techno-functional properties of black soldier fly larvae (BSFL) protein with the view to find alternative protein sources for food application. This study will extend current knowledge of the functionality changes of insect proteins due to the Maillard reaction.

## 2. Materials and Methods

### 2.1. Chemicals

All chemicals were purchased from Sigma-Aldrich (Aston Manor, South Africa). Prepared reagents were stored under conditions that prevented contamination or deterioration. The water used in the study was ultrapure water purified with a Milli-Q water purification system (Millipore, Microsep, South Africa).

### 2.2. BSFL Protein Extraction

Defatted BSFL flour (BSFL-DF) was mixed with Milli-Q water at a ratio of 1 : 10 (*w*.*w*^−1^), and the pH of the mixture was adjusted immediately to pH 10 using a 0.1 or 1 N NaOH solution. The mixture was stirred on a laboratory magnetic stirrer at a rate designed to prevent the formation of a vortex, for 30 min at ambient temperature (25°C ± 1.0). Subsequently, the samples were centrifuged at 10 000 *g* for 20 min at 4°C. Next, the pH of the supernatants was adjusted to 4.5 with 1 M HCl, and then, the suspension was left at 4°C overnight to facilitate protein precipitation. The precipitated proteins were recovered by centrifugation at 10 000 *g* for 30 min at 4°C. The BSFL protein concentrate was then freeze-dried and stored at -20°C for further analysis.

### 2.3. Synthesis of BSFL-Glu Conjugates, pH, and Browning Index

Black soldier fly larvae protein and glucose (BSFL-Glu) conjugates were prepared according to the method of Vhangani and Van Wyk [25] and Mshayisa [28] with slight modifications. BSFL protein concentrate and glucose (2 : 1 *w*.*w*^−1^) were dissolved in 100 mL of 0.1 M phosphate buffer at pH 9. The samples were transferred into 250 mL Schott bottles and heated at 50, 70, and 90°C in a water bath for 2, 4, 6, 8, and 10 h, respectively. After the heating period had elapsed, the resulting BSFL-Glu conjugates were immediately cooled in an ice bath. An unheated BSFL-Glu solution (0 min) was prepared as the control. A portion of the BSFL-Glu conjugates in solution was retained for pH measurements (pH meter: Metrohm, Switzerland). The remainder of the solutions were freeze-dried (Virtis, Wizard 2.0, NY, USA) and stored in airtight screw-capped glass bottles at -80°C until analysis. Before use, the powder was reconstituted to the required concentration with Milli-Q water and browning intensity was measured with a spectrophotometer (Lambda 25, Perkin Elmer, Singapore) at 294 and 420 nm. The pH and browning intensity (BI) measurements were used as nonspecific indicators of the MR.

### 2.4. Determination of Antioxidant Activity of BSFL-Glu Conjugates

#### 2.4.1. DPPH Radical Scavenging Activity

The DPPH radical scavenging (DPPH-RS) activity of BSFL-Glu conjugates was determined according to the method of Lertittikul et al. [28, 29] with slight modifications. A 0.12 mM solution of DPPH in ethanol was prepared daily and protected from light. A 4 mL aliquot of DPPH solution was added to 2 mL of BSFL-Glu conjugate (10 mg.mL^−1^) samples. The mixture was vortexed (Vortex-Genie 2, Scientific Industry Inc., USA) and allowed to stand at ambient temperature in the dark for 30 min. The absorbance of the mixtures was measured at 517 nm with a spectrophotometer (Lambda 25, Perkin Elmer, Singapore). A reaction mixture containing 2 mL distilled water and 4 mL ethanolic DPPH solution was used as control. All measurements were performed in triplicate. The percentage of DPPH-RS radical scavenging activity was calculated using the following equation:

(1) $$\begin{array}{c} \%\text{DPPH}-\text{RS}=1-\frac{A_{\text{sample }\left(517\text{nm}\right)}}{A_{\text{control }\left(517\text{nm}\right)}}\times 100, \end{array}$$

where *A*_control_ is the absorbance of the control at 517 nm and *A*_sample_ is the absorbance of the sample at 517 nm.

#### 2.4.2. ABTS^+^ Radical Scavenging Activity

The spectrophotometric analysis of ABTS^•+^ radical scavenging (ABTS-RS) activity of BSFL-Glu conjugates was determined according to a method as described by Yu et al. [28, 30] with slight modifications. The ABTS radical was prepared by reacting 7 mmol.L^−1^ ABTS solution and 2.45 mmol.L^−1^ potassium persulphate solution in equal volume, and the mixture was allowed to stand overnight in the dark at ambient temperature. The ABTS solution was diluted twenty-fold (20-fold) with Milli-Q water to obtain an absorbance of 1.5–1.6 at 730 nm. Fresh ABTS was prepared daily. A 4 mL aliquot of diluted ABTS solution was added to 200 *μ*L of aqueous BSFL-Glu conjugates solution (10 mg.mL^−1^), and the mixture was allowed to stand at room temperature for one hour. The absorbance was then measured at 730 nm using a spectrophotometer (Lambda 25, Perkin Elmer, Singapore). The control was prepared in the same manner with the substitution of distilled water for the sample. All measurements were performed in triplicate. The percentage of ABTS radical scavenging activity was calculated according to the following equation:

(2) $$\begin{array}{c} \%\text{ABTS}-\text{RS}=\frac{A_{\text{control }\left(730\text{ nm}\right)}-A_{\text{sample }\left(730\text{ nm}\right)}}{A_{\text{control }\left(730\text{ nm}\right)}}\times 100, \end{array}$$

where *A*_control_ is the absorbance of the control at 730 nm and *A*_sample_ is the absorbance of the sample at 730 nm.

#### 2.4.3. Reducing Power

The reducing power (RP) was determined according to the method of Vhangani and Van Wyk [25] and Mshayisa [28] with slight modifications. A one millilitre aliquot of each BSFL-Glu conjugates sample (10 mg.mL^−1^) was mixed with 2.5 mL of 0.2 M sodium phosphate buffer (pH 6.6) and 2.5 mL of 1.0% potassium ferricyanide. The reaction mixtures were incubated in a temperature-controlled water bath at 50°C (Memmert, Germany) for 30 min, followed by the addition of 2.5 mL of 10% trichloro-acetic acid after cooling at room temperature. The mixture was centrifuged at 1 750 g for 10 min at 25°C. The supernatant obtained (2.5 mL) was treated with 1 mL of Milli-Q water and 0.5 mL of 0.1% Ferric chloride. The absorbance of the reaction mixture was measured at 700 nm in a spectrophotometer (Lambda 25, Perkin Elmer, Singapore). All measurements were performed in triplicate. The RP was expressed as an increase in absorbance at 700 nm.

#### 2.4.4. Determination of Iron Chelation Activity

The Chelating activity of BSFL-Glu conjugates was determined according to the method of Gu et al. [31] with slight modifications. One millilitre BSFL-Glu conjugate sample (10 mg.mL^−1^) was mixed with 1.85 mL of Milli-Q water and 0.05 mL 2.0 mM FeCl_2_, and the mixture was allowed to stand at room temperature for 30 s. The reaction mixture thus obtained was added to 0.1 mL of 0.5 mM ferrozine and mixed; the absorbance was measured at 562 nm using a spectrophotometer (Lambda 25, Perkin Elmer, Singapore) after 10 min resting time and 5 min centrifugation at 3 000 *g*. The control was prepared in a similar manner, except that BSFL-Glu conjugates were replaced with Milli-Q water. All measurements were performed in triplicate. The percentage of chelating activity was calculated as follows:

(3) $$\begin{array}{c} \%\text{Chelating activity}=1-\frac{A_{\text{sample }\left(562\text{ nm}\right)}}{A_{\text{control }\left(562\text{ nm}\right)}}\times 100, \end{array}$$

where *A*_control_ is the absorbance of the control at 562 nm and *A*_sample_ is the absorbance of the sample at 562 nm.

### 2.5. Analysis of Techno-Functional Properties

#### 2.5.1. Foaming Property Evaluation

Foaming capacity (FC) and foam stability (FS) were determined according to the method of Zielińska et al. [32] with minor modifications. The freeze-dried BSFL-Glu conjugates sample was dispersed in distilled water (5% *w*.*v*^−1^) and centrifuged (Thermo Electron Corporation Jouan MR1812) at 10 000 rpm for 15 min. Then, 20 mL of the supernatant was homogenized in a high shear homogenizer mixer (Polytron PT 2500E) at a speed of 16 000 rpm for 3 min. The whipped sample was immediately transferred into a 50 mL cylinder. The total volume was read at time zero and 30 min after homogenization. The foaming capacity and foam stability were calculated from the formula:

(4) $$\begin{array}{c} \text{Foaming Capacity }\left(\text{FC}\right)=\frac{V_{a}-V}{V\;}\times 100,\\ \text{Foaming stability }\left(\text{FS}\right)=\frac{V_{30}}{V_{a}\;}\times 100, \end{array}$$

where *V* is the volume before whipping (mL), *V*_*a*_ is the volume after whipping (mL), and *V*_30_ is the volume after standing (mL).

#### 2.5.2. Emulsion Capacity and Stability of Conjugates

Emulsifying properties were determined according to the method of Coelho and Salas-Mellado [33] and Anzani et al. [34] with slight modifications. The sample was dispersed in distilled water (5% *w*.*v*^−1^) and centrifuged (Thermo Electron Corporation Jouan MR1812) at 9 000 rpm speed for 15 min; 15 mL of the supernatant was homogenized (Polytron PT 2500 E) with 15 mL of commercial sunflower oil at a speed of 18 000 rpm for 1 min. Next, the samples were centrifuged at 3 000 *g* for five minutes, and the volume of the individual layers was read. Emulsion stability was evaluated by heating the emulsion in a water bath set at 80°C for 30 min. Then, the samples were centrifuged at 3 000 *g* for five min. Emulsion capacity and emulsion stability were calculated from the formula:

(5) $$\begin{array}{c} \text{Emulsion capacity }\left(\text{EC}\right)=\frac{V_{\text{el}}}{V\;}\times 100,\\ \text{Emulsion stability }\left(\text{ES}\right)=\frac{V_{30}}{V_{\text{el}}\;}\times 100, \end{array}$$

where *V* is the total volume of tube contents, *V*_el_ is the volume of the emulsified layer, *V*_30_ is the volume of the emulsified layer after heating.

### 2.6. Data Analysis

All data were subjected to multivariate analysis of variance (MANOVA) using SPSS for Windows®, version 26.0 (IBM Corp, New York, USA) to ascertain whether the main effects resulted in significant differences in response variables. Duncan's multiple comparison post hoc test was used to test significant differences (*p* < 0.05) between individual means. Principal component analysis (PCA) was performed on aggregated mean centred data for antioxidant indices and techno-functional properties using Singular Value Decomposition (SVD) algorithm and cross-validation methods with 18 segments to determine potential clusters with the use of The Unscrambler software, version 11 (CAMO Software, Oslo, Norway). Graphs and figures were generated using the Origin software, version 9.60 (Origin labs, Northampton, MA, USA) and The Unscrambler software.

## 3. Results and Discussion

### 3.1. The Extent of Maillard Reaction

**Figure 1:**
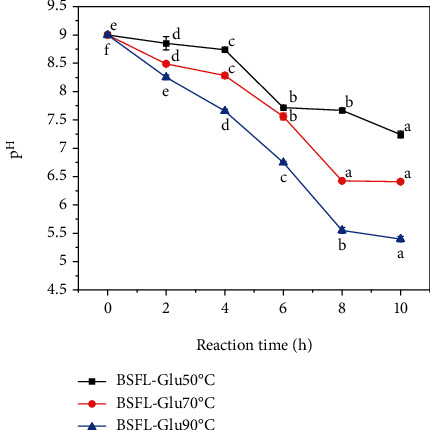
Changes in pH of BSFL-GLu conjugates model system as a function of reaction time. Values are mean ± standard deviation; means with different superscript are significantly different at the same heating temperature.

During the Maillard reaction (MR), the initial reactants are consumed which consequently results in the formation of initial, intermediate, and advanced brown polymers. The initial condensation step of the MR is facilitated by higher pH values, and thus, it is vital to monitor the pH of the model system. Therefore, change in pH values during the MR was monitored to evaluate its significance during the reaction. The pH of BSFL-Glu conjugate model systems at 50, 70, and 90°C as a function of reaction time is shown in Figure 1. For each model system, the MANOVA with Duncan's multiple range tests revealed that the decrease in pH observed as the reaction temperature and time increased was significant (*p* < 0.05). This indicated that the largest pH reduction resulted from higher reaction temperature and time combinations. As shown in Figure 1, the final pH values of BSFL-Glu conjugates reacted at 90°C were lower than those at 50°C and 70°C, respectively. These results are due to the fact that the degree of the Maillard reaction was greater at high temperatures than at low temperatures. Vhangani and Van Wyk [25] also observed a decrease in pH of MRPs from fructose-lysine and ribose-lysine model systems. Moreover, Benjakul et al. [35] also reported a decrease in pH of porcine plasma protein-sugar model systems heated up to 5 hours. In the early stages of the reaction, the decrease in pH can be due to the formation of formic and acetic acid during the MR [36, 37] and the free amino groups of BSFL protein consumed. Additionally, higher pH was further away from the BSFL protein concentrate's isoelectric point and could therefore induce stronger intramolecular electrostatic repulsions that may lead to more extensive unfolding, more exposed reactant amino groups, and greater solubility, both of which contribute to improving the conjugation reaction.

### 3.2. Browning Intensity

**Figure 2:**
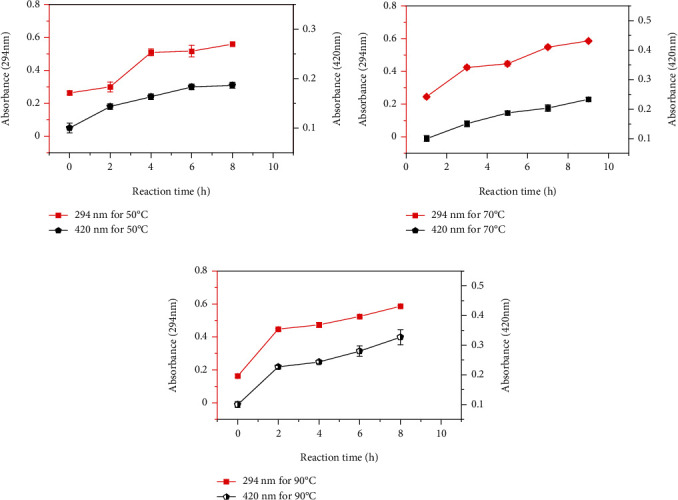
Changes in absorbance at 294 nm and 420 nm of BSFL-Glu conjugates as a function of reaction time: (a) 50°C, (b) 70°C, and (c) 90°C.

The UV-VIS absorption of Maillard reaction products (MRPs) at 294 nm and 420 nm has been generally accepted as nonspecific markers to evaluate the formation of Amadori compounds at the early-intermediate stage and melanoidins at the final stage. In the early stage of the MR, intermediate compounds are formed. The early to intermediate stage Amadori reaction products with characteristic absorption at 294 nm and advanced stage melanoidins which represent brown pigments at 420 nm are usually measured as an indicator of the extent of the MR in model systems. As shown in Figures 2(a)–2(c), a gradual increase in absorbance at 294 nm was observed as the reaction time increased at the same reaction temperature up to 10 h, indicating that the increase of the reaction time increases the formation of intermediate Maillard reaction products. BSFL-Glu conjugates prepared at higher reaction temperature (90°C) exhibited the highest increase (*p* < 0.05) in absorbance as a function of reaction time. The absorbance at 294 nm was used to determine the intermediate compounds of the MR [35]. From these results, the increase in absorbance at 294 nm, irrespective of heating temperature, suggests that the early intermediate compounds were dominant in all BSFL-Glu conjugates.

The easiest observable result of the Maillard reaction is brown colour production (A420 nm) since it gives a visual estimate. Its magnitude is also used in foods as a measure of the degree to which the Maillard reaction occurs and signifies an advanced stage of the Maillard reaction [38]. As the heating time increased (*p* < 0.05) irrespective of the heating temperature (Figures 2(a)–2(c)), an increase in browning of BSFL-Glu MRPs as measured by absorbance at 420 nm was observed. The increase in absorbance at 420 nm indicates the development of browning pigment in the final stage of the Maillard reaction [39]. The reaction consists of the condensation of an amino compound and sugar fragments into polymerised glycoproteins and the brown pigment melanoidin. As evidenced by the increased absorption at 294 nm, the increase in brown pigment production was coincidental with an increase in colourless intermediate formation, which indicates that brown pigments were developed proportionally with the intermediate products produced (Figures 2(a)–2(c)). These findings suggest that, under mild incubation conditions, the formation of intermediate Amadori compounds takes place prior to coloured melanoidins.

## 4. Antioxidant Properties

According to Vhangani and Van Wyk [40], it is vital to implement more than one type of assay to evaluate the antioxidant activity of the products formed during the Maillard reaction. This is due to the complexity and nature of the myriad of the formed intermediate compounds which have not yet been fully elucidated. Thus, the antioxidant activity of heat-treated BSFL-Glu conjugates was assayed in terms of their free radical-scavenging activity, reducing power and metal chelation.

### 4.1. DPPH Radical Scavenging

**Figure 3:**
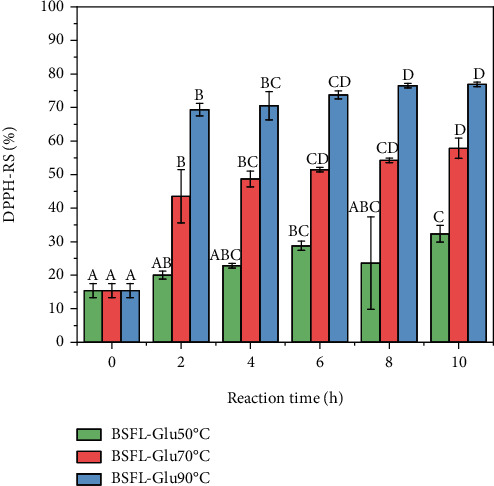
DPPH radical scavenging activity of BSFL-Glu conjugates as a function of reaction temperature. Values are mean ± standard deviation; means with different superscripts are significantly (*p* < 0.05) different at the same heating temperature.

The DPPH-RS activity indicates the hydrogen-donating ability of antioxidants. The DPPH-RS of BSFL-Glu conjugates is presented in Figure 3. In general, the DPPH-RS of BSFL-Glu conjugates increased significantly (*p* < 0.05) with an increase in reaction time at 50, 70, and 90°C, respectively. At 50°C, the DPPH-RS of the conjugates ranged from 15.47 to 32.37%, and it was significantly higher than the control (*t* = 0). Compared with the lower temperatures (50 and 70°C), conjugates produced at 90°C had a significantly higher DPPH radical scavenging activity (*p* < 0.05). BSFL protein concentrate heated alone exhibited a weak radical scavenging activity with only 3% radical activity and there was no significant difference (*p* > 0.05) over the reaction conditions (time and temperature) examined in this study (results not shown). This signifies that BSFL protein conjugated with glucose can significantly improve the antioxidant activity of BSFL protein.

The results of this study are in agreement with Jiang et al. [41] who observed an increase in DPPH-RS of the tripeptide Ile-Pro-Pro (IPP) MRPs as a function of reaction time. Moreover, ultrafiltered casein-glucose MRPs model systems showed strong DPPH radical scavenging [42]. Taken collectively, the results of this study show that conjugates prepared from insect protein concentrate extracted from BSFL possess hydrogen-donating capacity, which implies that these conjugates exhibit potency to react with free radicals. This opens up new avenues or possibilities for the application of insect proteins as novel functional ingredients in food formulations.

### 4.2. ABTS Radical Scavenging

**Figure 4:**
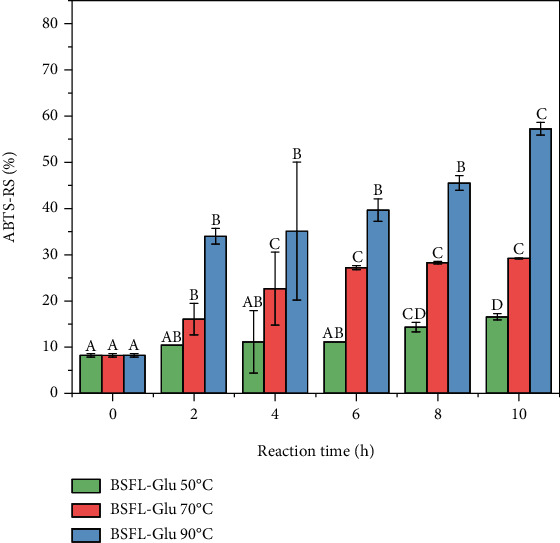
ABTS radical scavenging activity of BSFL-Glu MRPs as a function of reaction temperature. Values are mean ± standard deviation; means with different superscript are significantly different at the same heating temperature.

The ABTS^+^ radical scavenging activity was determined in order to assess the antioxidant potential of BSFL-Glu conjugates. As depicted in Figure 4, the ABTS^+^ radical scavenging activity of conjugates produced at 50°C ranged from 10.5 to 16.5% and exhibited the lowest ABTS radical scavenging activity. This can be attributed to the electron-donating ability of some of the amino acids of BSFL protein, which reduces ABTS^+^ radicals to offer weak antioxidant activity. However, increasing the temperature to 70°C increases significantly (*p* < 0.05) the ABTS^+^ radical scavenging activity as a function of reaction time. The results of this study concur with You et al. [36] who reported that the ABTS^+^ radical scavenging activity of a silver carp protein hydrolysate-glucose system obtained at 60°C was significantly higher than that obtained at 50°C.

The ABTS^+^ radical scavenging activity of BSFL-Glu conjugates produced at 90°C exhibited significantly (*p* < 0.05) higher scavenging activity as a function of reaction time (Figure 4). Sun et al. [43] reported that the MRPs formed by *α*-lactalbumin and different types of reducing sugars exhibited greater ABTS-RS than native *α*-lactalbumin. The browning compounds formed during the Maillard reaction, which are primarily composed of melanoidins, have been reported to be significant contributors to the radical scavenging ability [36, 44]. The results of this study strongly confirm that glycation could induce the antioxidant activity of the edible insect protein, the intensity of which depends on the duration of reaction time and temperature. To the best of our knowledge, this is the first study to explore the effect of reaction temperature and time on the antioxidant properties of BSFL-Glu conjugates.

### 4.3. Changes in Reducing Power

Reducing power has been used to determine the antioxidant effect of MRPs, and it is regarded as a good indicator of antioxidant activity of food components [45]. This assay measures particularly the antioxidant activity of MRP conjugates with the hydroxyl groups of conjugates playing a role in the reducing activity through their redox potential of transferring electrons [25, 45]. In this assay, in the presence of antioxidants, the ferric chloride/ferricyanide complex is reduced to the ferrous form (Fe^2+^), and the Fe^2+^ concentration can, therefore, be assessed spectrophotometrically by measuring the Prussian blue colour of Perl at 700 nm. The reducing power of BSFL-Glu conjugates increased significantly (*p* < 0.05) with an increase in reaction time, with conjugates derived at 50°C and 70°C (Table 1) having the lowest and highest reducing power, respectively. Heat-induced MRPs from ribose-lysine [25], glucose-glycine [46], and porcine plasma protein-glucose [29, 35] model systems also possessed reducing power. From these results, the reducing power of BSFL-Glu conjugates increases as a function of reaction time at all temperatures and this correlated well with browning intensity (Figure 2). The increase in reducing power at higher temperatures (90°C) can be ascribed to the fact that the protein structure was unravelled (denatured) at elevated temperatures, enhancing the Maillard reaction rate, and producing more intermediate and advanced products, with enhanced reducing power (Table 1). Moreover, the results of DPPH-RS and ABTS-RS assays together with the reducing power results indicated that the difference in the antioxidant activity of the conjugates from this edible insect protein was mainly attributable to the mechanism of single electron transfer (SET). Conjugation of BSFL protein with glucose via the Maillard reaction may induce structural changes in the BSFL-Glu system, which result in the formation of products or compounds that contribute to the reducing power.

### 4.4. Fe^2+^ Chelating Activity

**Figure 5:**
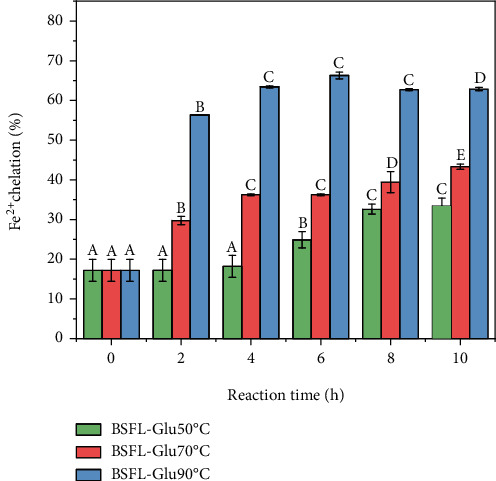
Iron chelating activity of BSFL-Glu conjugates as a function of reaction time. Values are mean ± standard deviation; means with different superscript are significantly different at the same heating temperature.

The generation of reactive oxygen species such as hydroxyl radical (^•^OH) and superoxide anion (O_2_^•−^) can be catalysed by transition metal ions, such as Fe^2+^ and Cu^2+^. Fe^2+^, in particular, generates the hydroxyl radical through the Fenton reaction that accelerates the reaction of the lipid peroxidation reaction. Chelators can form complexes with metal ions and inhibit the Fenton reaction. Consequently, the chelation of metal ions contributes to antioxidant activity. MRPs are known metal chelators [25, 28, 31], and their metal-ion binding affinity has been proposed as a possible mechanism to explain their antioxidant activity [47] because transition metals, especially iron and copper, are involved in the generation of free radicals by Fenton reaction. The chelating activity of BSFL-Glu MRPs is shown in Figure 5. The chelating activity of BSFL-Glu conjugates increased significantly (*p* < 0.05) as a function of reaction time and temperature. Chelating activity significantly increased from 17.20 to 28.20% for BSFL-Glu conjugates heated at 50°C.

Generally, BSFL-Glu conjugates heated at 90°C exhibited higher metal chelation activity compared to those at 50°C and 70°C. For the metal chelating activity of BSFL-Glu conjugates at 90°C, a significant increase was observed until maximum (64.45%) at 6 h; this was then followed by a slight decrease until the end of the heating period. This implies that conjugates produced at 90°C for 6 h have the ability to inhibit lipid oxidation. Dong et al. [48] thought that the significant decrease in metal chelation of casein-glucose conjugates after 6 h could be partially due to the loss of free amine groups during thermal treatment. The results of this study coincide with the findings of Mshayisa [28] and Zeng et al. [23]. This decrease of metal chelation after 6 h can be ascribed to the formation of compounds with a high molecular weight at higher reaction temperatures and time. The results of this study are in agreement with Gu et al. [42], who observed high metal chelation of MRPs with a higher molecular weight in casein-glucose model systems. The observed chelating activity can be due to the hydroxyl groups originating from BSFL-Glu conjugates. Metal chelation activity plays an imperative role in antioxidant activity as it results in reducing the concentration of the transition metal which catalyses lipid oxidation [49, 50].

## 5. Techno-Functional Properties

### 5.1. Foaming Capacity and Stability

**Figure 6:**
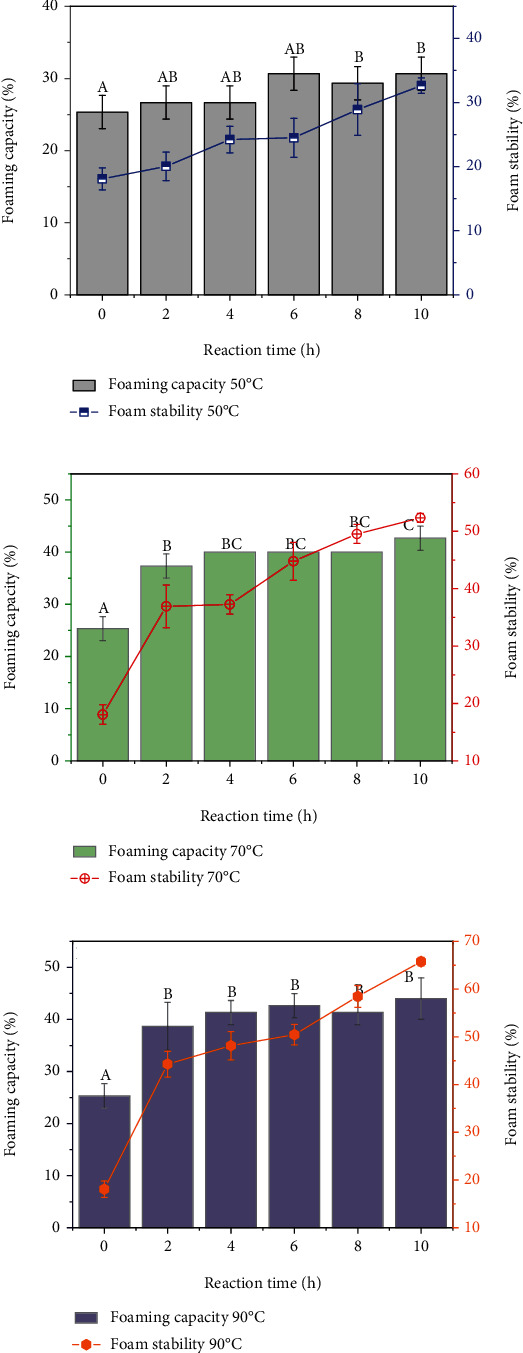
Foaming capacity and foam stability of BSFL-Glu conjugates as function of reaction time: (a) 50°C, (b) 70°C, and (c) 90°C. Values are mean ± standard deviation; means with different superscripts are significantly different at the same heating temperature.

For items such as meringues, sponge cake, marshmallows, or ice creams, foaming properties are an essential techno-functional property of food protein ingredients. Therefore, knowledge of the foam capacity and stability of glycated edible insect protein is crucial in the quest for novel ingredients to be incorporated in food products. Figures 6(a)–6(c) illustrates the percentage of foam capacity (FC) and foam stability (FS) of conjugates derived from BSFL-Glu conjugates at 50°C, 70°C, and 90°C. All BSFL-Glu conjugates had the ability to form foams. In the case of BSFL-Glu conjugates heat-treated at 50°C (Figure 6(a)), the FC was not significantly (*p* > 0.05) different between 2 and 4 h. The FC increased significantly (*p* < 0.05) after 6 h to a maximum of 30.67% and then decreased. However, the FS increased (*p* < 0.05) gradually during the heating period to a maximum of 32.64%. Jian et al. [15] reported a decrease in foaming capacity of bovine serum albumin (BSA) conjugated with glucose at 45°C. This apparent contradiction may be attributed to the differences in the protein hydrophobicity in the respective studies.

The foaming capacity of BSFL-Glu conjugates produced at 70°C showed a significant gradual increase (*p* < 0.05) from 2 h to 10 h (Figure 6(b)). Furthermore, the FS increased after 6 h to 42.66%. The conjugates prepared at 70°C performed better in terms of FC and FS compared to those at 50°C. BSFL-Glu conjugates treated at 90°C for 10 h showed superior foaming capacity (44%) compared to the conjugates heated for shorter times. A similar trend was also observed in the foam stability at 90°C (Figure 6(c)). This can be attributed to the formation of a viscoelastic thick and dense layer around the entrapped air bubble interface and preventing the foam from collapsing. In general, the results of this study reveal that the foams derived from BSFL-Glu conjugates heated for longer periods (e.g. 10 h) were considerably more stable, compared to those obtained at shorter reaction times, irrespective of the temperature. This is due to the modulation of the hydrophilic-lipophilic balance of proteins and the creation of conjugates with the inherent capability to entrap air bubbles during foam formation and retain them for longer periods by the Maillard reaction. These results were consistent with several previous reports showing that foaming capacity increased after glycation [20, 51, 52] due to changed hydrophobicity and conformation after glycation. This work further demonstrates that glycation improved the techno-functional properties of proteins. Thus, modified BSFL protein via glycation could be used as a functional ingredient in food applications where foaming properties are desirable.

### 5.2. Emulsion Capacity and Stability

**Figure 7:**
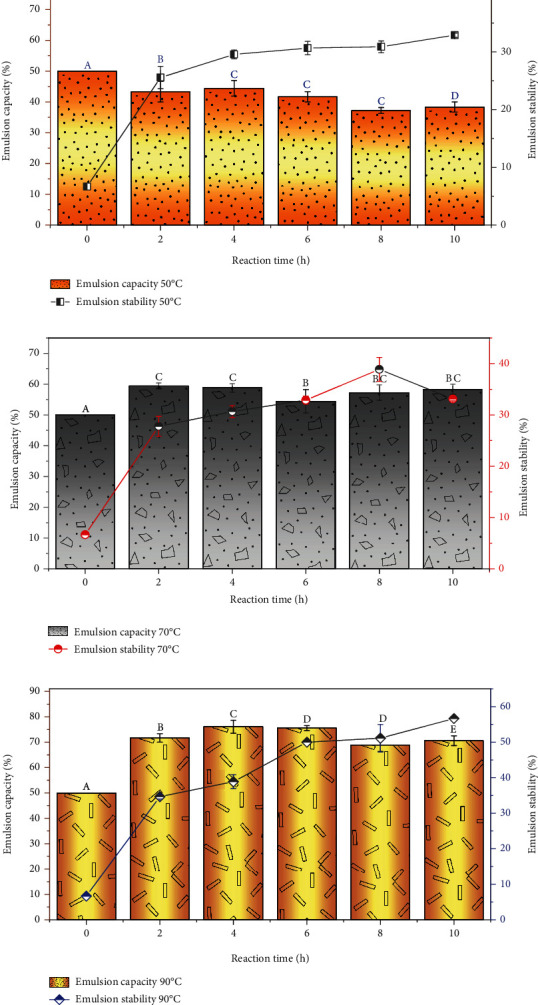
Emulsion capacity and stability of BSFL-Glu conjugates: (a) 50°C, (b) 70°C, and (c) 90°C. Values are mean ± standard deviation; means with different superscript are significantly different at the same heating temperature.

In food systems, emulsifying properties play an important role since they directly contribute to the texture and the organoleptic properties of food. They are also essential ingredients in the formation of structures in various food products, such as mayonnaise and dressings, coffee creamers, gravies, cream liqueurs, some fruit drinks, and many meat products. The emulsion capacity and stability of BSFL-Glu conjugates are depicted in Figures 7(a)–7(c). The emulsifying capacity of BSFL-Glu conjugates derived at 50°C decreased significantly (*p* < 0.05) as the reaction time increased. The control (*t* = 0) had a significantly higher (*p* < 0.05) emulsion capacity compared to the conjugates. However, emulsion stability increased significantly (*p* < 0.05) as the reaction time increased. These observations were inconsistent with those reported by Rangsansarid et al. [53], namely, that no improvements in emulsion stability were observed for BSA-sugar (glucose, allose, and 6-O-octanoyl-D- glucose) conjugates. On the contrary, BSFL-conjugates prepared at higher temperatures, for example, 70 and 90°C had significantly higher emulsion capacity compared with the control (*t* = 0). The emulsion capacity of BSFL-Glu conjugates at 70°C ranged from 54.44 to 59.45%. The emulsion stability increased significantly (*p* < 0.05) as a function of reaction time, with conjugates produced after 8 h exhibiting the highest emulsion stability (35.89%). In general, the emulsion stability of the conjugates increased significantly (*p* < 0.05) as a function of time, irrespective of glycation temperature. The reduction in interfacial tension by the emulsifier is mainly achieved by directing the hydrophobic and hydrophilic parts of the emulsifier toward the nonpolar fraction (oil phase) and the polar component (water phase), respectively. To the knowledge of the authors, the data presented in Figures 7(a)–7(c) is a first for BSFL-based conjugates and thus enriches the current literature on the potential incorporation of edible insect-derived ingredients in the food supply chain.

In this study, the BSFL-Glu conjugated at 90°C showed higher emulsion capacity and stability due to the Maillard reaction. This increase can be attributed not only to an increase in the reactivity of the BSFL protein structure between the carbonyl group and the amino group but also to a greater unfolding of the BSFL protein structure, showing a higher number of reactive functional groups (lysine residues), favoured by high temperatures. Most notably, this is the first study, to our knowledge, to examine the effect of Maillard conjugation on insect protein, in particular black soldier fly larvae. The high emulsion stability and capacity of BSFL-Glu conjugates suggest that they have the potential to be used as novel functional ingredients in food processing.

## 6. Principal Component Analysis

### 6.1. Principal Components Explaining the Variability in BSFL-Glu Browning Index and Antioxidant Activity

**Figure 8:**
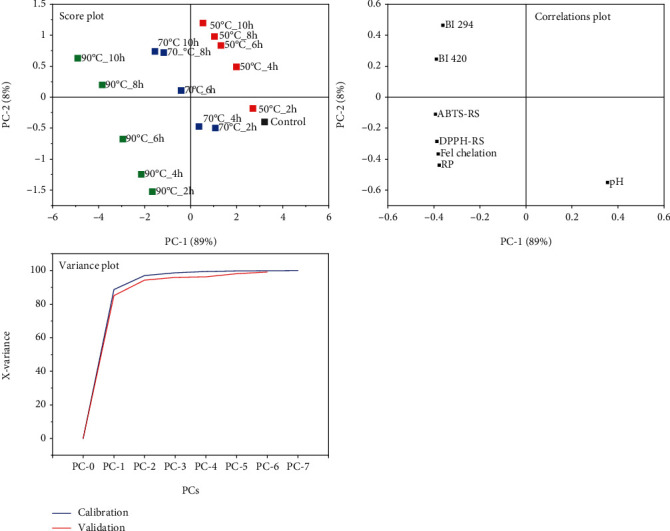
PCA plots of independent (scores) and dependent (loadings) variables for BSFL-Glu antioxidant indices.

Principal component analysis (PCA) was used to reduce the variability of data among the heat-treated conjugates. In principal component analysis, the original data matrix is converted to loadings and scores (tested parameters) vectors, whereby new variables—the principal components—were obtained. PCA was, therefore, applied to the browning indicators, pH reduction, and antioxidant properties of BSFL-Glu conjugates. The PCA exhibiting score, correlation loadings, explained variance, and influence plots are shown in Figure 8. The explained variance plot gives an indication of how much variation in the data is described by the components. In this study, there is no clear break in the explained variance plot, which is the calibration is closer to the validation plot. The variability in the data could be explained by two PCs where PC1 contributes 89% of the variability and PC2 contributes 8% while PC3 explains 2%. The cumulative variation explained by PC1 and PC2 was 97% (Figure 8). Therefore, the first two PCs are sufficient to describe the variability in terms of BSFL-Glu conjugates in terms of the browning index and antioxidant properties.

Score plots were used for outlier identification, identification of groups or trends, exploration, of replicate similarities and more. The score plot showed that PC1 describes the heating temperature since samples are distributed from right to left according to the heating temperatures. Clusters of BSFL-Glu as a function of heating temperature could be clearly observed from the score plot. Moreover, the correlation loading matrix (Figure 8) shows that the outer ellipse explained 100% of the variability and while the inner ellipse indicates 50% of the explained variance. As can be seen from Figure 8 (top right), the browning index and antioxidant properties (DPPH-RS, ABTS-RS, Fe chelation and reducing power) were close to each other reflecting a high positive correlation with each other and overwhelmingly explained the PC1 direction. The pH reduction was diametrically opposed to the browning index and antioxidant properties. Therefore, these two components (PC1 and PC2) would be adequate for the prediction of browning and antioxidant indices of BSFL-Glu conjugates.

### 6.2. Principal Components Explaining the Variability in BSFL-Glu Techno-Functional Properties

**Figure 9:**
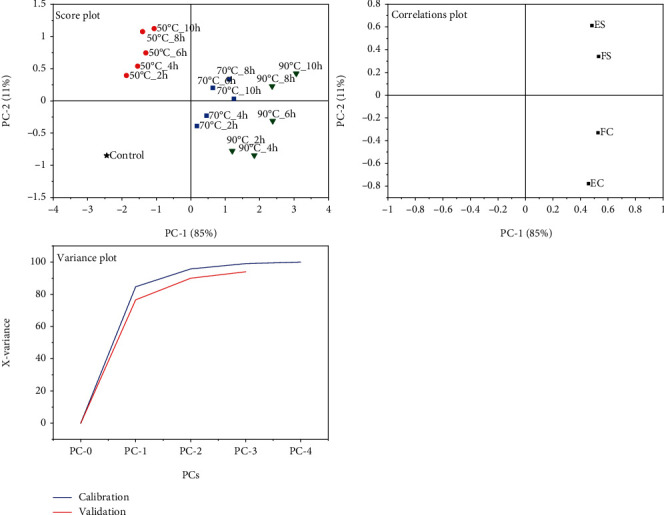
PCA plots of independent (scores) and dependent (loadings) variables for techno-functional properties.

Principal component analysis was further used to determine the clusters or sample groupings concerning the techno-functional properties. From the explained variance plot, it can be deduced that two PCs are sufficient to explain the observed variance (Figure 9). The score plot indicates that cumulative variance explained by PC1 and PC2 were 85% and 11%, respectively, with a total of 96%. PC1 showed positive loadings in FC and FS, while PC 2 showed negative loading for EC. The PCA confirms the results obtained with regard to the heating temperature. From the model applied, the BSFL-Glu samples could be clearly separated into clusters based on the heating temperature.

## 7. Conclusion

The effect of heating temperature and time on the antioxidant and techno-functional properties of BSFL-Glu conjugates was demonstrated in the current study. Browning intensity, DPPH-RS and ABTS-RS radical scavenging activity consistently increased with an increase in reaction temperature and time. BSFL-Glu conjugates exhibited enhanced techno-functional properties, especially foaming stability and emulsion stability. The findings presented to demonstrate the use of conjugation through the Maillard reaction as an approach to enhance the functionality of proteins, which is particularly applicable to highly sought-after edible insect proteins. The performance of the BSFL-Glu conjugates in applications such as foams and emulsions need to be explored in future studies via more time-temperature combinations, since it was shown that heating time and temperature influence the extent of the glycation reaction. The results of this study indicated that protein conjugates of edible insects (BSFL) have significant potential for use as functional ingredients for the development of an effective foam and emulsion structure. Nevertheless, the effects on these conjugates with variation in pH and salts need to be examined in future work.

## Figures and Tables

**Table 1:** Changes in reducing power of BSFL-Glu conjugates as a function of reaction time and temperature (700 nm).

Reaction time (h)	Reaction temperature		
	50°C	70°C	90°C
0	0.02 ± 0.01^a^	0.12 ± 0.01^a^	0.41 ± 0.03^a^
2	0.04 ± 0.00^b^	0.33 ± 0.02^b^	0.76 ± 0.04^b^
4	0.05 ± 0.00^b^	0.34 ± 0.01^b^	0.80 ± 0.03^b^
6	0.06 ± 0.00^c^	0.38 ± 0.01^c^	0.83 ± 0.03^b,c^
8	0.07 ± 0.01^d^	0.41 ± 0.01^d^	0.81 ± 0.04^b^
10	0.11 ± 0.01^e^	0.42 ± 0.01^d^	0.89 ± 0.06^c^

^a^Values are the mean ± SD (*n* = 3); values with different superscript in the same column indicate significant differences (*p* < 0.05).

## Data Availability

Data can be provided upon request.
